# Rational Adaptation in Lexical Prediction: The Influence of Prediction Strength

**DOI:** 10.3389/fpsyg.2021.622873

**Published:** 2021-04-14

**Authors:** Tal Ness, Aya Meltzer-Asscher

**Affiliations:** ^1^Sagol School of Neuroscience, Tel Aviv University, Tel Aviv, Israel; ^2^Linguistics Department, Tel Aviv University, Tel Aviv, Israel

**Keywords:** prediction, adaptation, language processing, bayesian adaptation, prediction error

## Abstract

Recent studies indicate that the processing of an unexpected word is costly when the initial, disconfirmed prediction was strong. This penalty was suggested to stem from commitment to the strongly predicted word, requiring its inhibition when disconfirmed. Additional studies show that comprehenders rationally adapt their predictions in different situations. In the current study, we hypothesized that since the disconfirmation of strong predictions incurs costs, it would also trigger adaptation mechanisms influencing the processing of subsequent (potentially) strong predictions. In two experiments (in Hebrew and English), participants made speeded congruency judgments on two-word phrases in which the first word was either highly constraining (e.g., “climate,” which strongly predicts “change”) or not (e.g., “vegetable,” which does not have any highly probable completion). We manipulated the proportion of disconfirmed predictions in highly constraining contexts between participants. The results provide additional evidence of the costs associated with the disconfirmation of strong predictions. Moreover, they show a reduction in these costs when participants experience a high proportion of disconfirmed strong predictions throughout the experiment, indicating that participants adjust the strength of their predictions when strong prediction is discouraged. We formulate a Bayesian adaptation model whereby prediction failure cost is weighted by the participant’s belief (updated on each trial) about the likelihood of encountering the expected word, and show that it accounts for the trial-by-trial data.

## Introduction

Despite the seemingly inexhaustible capabilities of the human brain, cognitive research has shown time and again that in some respects, our processing resources are limited. For example, although our brain can store over 10^9^ bits of information over our lifetime (Von Neumann, 1958), the processing of visual objects or linguistic input is limited to no more than a few items at once (e.g., “the magical number seven” suggested by Miller, 1956, “the magic number four,” Cowan, 2010; Green, 2017, or even fewer items, as suggested by McElree, 2001). It is therefore often assumed (explicitly or implicitly) that successful language processing requires efficient resource allocation.

One core aspect of language processing, which may seem somewhat contradictory to this assumption, is prediction. Over the past decades, accumulating evidence provided strong support for the idea that during language processing, we engage in actively anticipating upcoming input, rather than passively waiting for the input in order to process it as it unfolds. This anticipatory processing is evidenced in reduced processing difficulty for predictable relative to unpredictable words, manifested in reduced reading times or reaction times (RT; Ehrlich and Rayner, 1981; Schwanenflugel and Shoben, 1985; Traxler and Foss, 2000) and reduced amplitudes of the N400 event-related potentials (ERP) component (e.g., Kutas and Hillyard, 1984; DeLong et al., 2005; Wlotko and Federmeier, 2012). Notably, evidence suggests that this anticipation of upcoming input is, at least under certain circumstances, as specific as predicting the exact word that is expected to appear, including its phonological form, grammatical gender, etc. (e.g., Wicha et al., 2004; DeLong et al., 2005; van Berkum et al., 2005; Martin et al., 2013; Nieuwland et al., 2018; Nicenboim et al., 2019; Szewczyk and Wodniecka, 2020). For example, Wicha et al. (2004) examined ERPs elicited when Spanish native speakers read a determiner (el/la, *un/una*, and *las/los*), which appears prior to the noun and has to agree with the noun’s grammatical gender. Their results show that in sentences that lead to a highly probable noun, determiners with a gender feature that does not match the predictable noun elicit enhanced positivity. These results indicate that the predictions generated were beyond the conceptual level, such that the specific noun was predicted, including its grammatical features.

Allocating resources to generate predictions, especially such specific predictions, intuitively seems to be a very wasteful processing strategy. We use language to communicate information, and in order for an utterance to be informative it has to be unpredictable to some extent (i.e., no new information would be gained by the listener, if they had, in advance, all the information needed in order to predict the utterance with 100% certainty prior to perceiving it). Why, then, generate predictions that will inevitably have some likelihood of being incorrect, when we can instead merely process the input as it is perceived? This question becomes even more puzzling when taking into account evidence of prediction failure costs. Predictability is often measured using the cloze task, in which participants are given the beginning of a sentence or a phrase, and are asked to provide the first completion that comes to mind. From this task, the predictability of a word is reflected in the word’s cloze probability, defined as the proportion of participants who provided this word as a completion. Additionally, the constraint of a context is also calculated, defined as the cloze probability of it most common completion. It is considered to reflect the extent to which the context can lead to a strong prediction. Recent studies indicate that the processing of an unexpected (low cloze probability) word entails additional costs when it is presented in a high constraint sentence, i.e., when the initial prediction was strong. These costs are not incurred when processing a similarly unexpected word if no strong prediction was formed in the first place. This increased difficulty is mostly evidenced in the frontal post-N400 positivity (f-PNP), an ERP component that is elicited by unexpected words, only when a highly probable prediction was initially available (e.g., Federmeier et al., 2007). Since these costs are not incurred by unexpected words in low constraint contexts, they cannot be attributed to the processing of an unexpected word in and of itself. They are therefore attributed to the need to handle the incorrect prediction. This prediction failure cost was suggested to stem from a commitment made to the initial (strong) prediction, requiring its inhibition or suppression in order to integrate the actual input (e.g., Kutas, 1993; Ness and Meltzer-Asscher, 2018a,b; Kuperberg et al., 2020). We note that such inhibition can be needed at different levels of representation, namely, prediction failure costs can be incurred by a need to inhibit a low-level representation of the word in the lexicon, a higher-level representation of the sentence or the message, or both (see further discussion of this distinction in the General discussion). However, regardless of the specific nature of prediction failure costs, engagement in prediction is “wasteful” in processing resources not only due to the resources needed for the generation of predictions, but also due to the resources needed to handle the disconfirmation of strong predictions.

Several reasons have been suggested for the use of prediction as a language processing strategy despite its “wastefulness,” explaining why engaging in prediction constitutes a sensible use of resources after all. For example, prediction may be helpful in reducing the ambiguity that exists in most linguistic input, either due to semantically/grammatically ambiguous utterances or due to perceptual ambiguity (e.g., arising from noisy input and production variation), by constraining the interpretation of the input to more probable meanings/representations. Additionally, prediction has been suggested to provide an effective learning mechanism based on prediction error signals. It has also been argued to enable coordinated “turn taking” during dialog (for discussion of motivations for prediction see Huettig, 2015). Prediction thus serves important functions, meaning that allocating resources for prediction is not inefficient. Notably, however, even though in general it is presumably useful to engage in prediction, the mere fact that prediction bears costs means that situations can differ in how beneficial prediction is. For example, if prediction is indeed helpful in disambiguating perceptually ambiguous input, then it may be more effective to allocate resources to generate strong predictions in a noisy environment than in a quiet one. If prediction is needed to coordinate “turn taking,” we may engage more in prediction during a conversation than during passive listening (e.g., listening to a lecture or watching a movie). Moreover, regardless of the specific reason(s) that make prediction a useful processing strategy, the costs of prediction failure may outweigh the benefits derived from successful predictions, in a situation where unexpected input is often encountered. Hence, while it is reasonable to allocate resources for prediction, it is inefficient to always do so to the same extent, regardless of the situation.

Indeed, several previous studies have shown that prediction can be adapted to different situations (e.g., Neely, 1977; Schwanenflugel and Shoben, 1985; Hutchison, 2007; Lau et al., 2013; Brothers et al., 2017, 2019). Most commonly this is demonstrated as a relatedness proportion effect, i.e., the facilitation due to relatedness between a prime and a target in a prime-target lexical/semantic decision task increases when the proportion of related prime-target pairs increases (e.g., Lau et al., 2013). This indicates that when the prime and the target are often related, participants increase their reliance on semantic relatedness to the prime word in anticipating the target word (this maximization of the use of contextual information can be explained by several frameworks, in particular relevance theory, see Sperber and Wilson, 1996). Recently, this adaptation was shown to fit a Bayesian model, in which participants repeatedly update their belief about the likelihood of a related prime-target pair, and this belief is used in order to weigh the relative influence of relatedness, relative to general word frequency (Delaney-Busch et al., 2019). Namely, when the likelihood of a related prime-target pair is low, participants do not adopt a prediction strategy, and reaction times are mostly influenced by word frequency; then, as participants accumulate evidence of a high likelihood of relatedness between primes and targets, they adopt a prediction strategy that relies more on semantic relatedness, and these predictions become stronger the greater the participant’s belief that related prime-target pairs are likely to appear.

Thus, prediction requires processing resources, different situations differ in how beneficial prediction is and what the optimal prediction strategy is, and evidence suggests that we have means to adapt our prediction mechanisms accordingly. This state of affairs poses two questions:

- When do comprehenders alter their prediction strategies (i.e., can other factors, besides proportion of related prime-target pairs, trigger changes to prediction strategies)? Specifically, the current study aims to test whether comprehenders alter their prediction strategies when they experience failure of strong predictions.
- How do comprehenders optimize their prediction strategies (i.e., which processes or mechanisms are susceptible to transient changes, and what are these changes)? Specifically, the current study aims to test whether comprehenders can alter their tendency to commit to strong predictions, in order to achieve an optimal balance between the benefits of successful prediction and the costs of prediction failure.

Thus, the current study focuses on the role of prediction strength and prediction failure in adaptation of prediction. As discussed above, the disconfirmation of strong predictions incurs prediction failure costs associated with a need to inhibit the falsely predicted word, due to some form of commitment made to the strong prediction. Thus, in the current study, we hypothesized that the disconfirmation of strong predictions serves as a trigger for adaptation, and that this adaptation influences subsequent predictions by decreasing the tendency to commit to strong predictions, in order to avoid prediction failure costs.

A previous study provides indication that prediction failure costs can be affected by adaptation. Schwanenflugel and Shoben (1985) have conducted a series of experiments, which showed that prediction failure costs were increased when a participant encountered a large proportion of high constraint sentences in which the most predictable word appeared, but not when they encountered a large proportion of low constraint sentences in which the most predictable word appeared. This indicates that repeated confirmation of predictions leads to increased costs when a prediction is disconfirmed. Notably, in this study, the manipulation was conducted by the addition of fillers, which were high\low constraint trials in which the most predictable word is presented (keeping constant the number of trials in which an unexpected word appeared instead of the predicted word). Namely, in this experiment, successful predictions served as the trigger for adaptation. Thus, this study indicates that prediction failure costs are influenced by adaptation, but not that prediction failure can serve as a trigger for adaptation.

Additionally, this design does not allow to isolating the contribution of prediction strength to this adaptation. Trials in which the most predictable word is presented in a high vs. low constraint inevitably differ not only in the constraint of the context, but also in the cloze probability of the presented word, since the most predictable word in low constraint contexts is not as predictable as the most predictable word in high constraint contexts. As inherent to the definition of cloze probability, a word with 80% cloze probability was provided as the first completion that came to mind by 80% of the participants in the cloze task, reflecting that it would likely be the strongest prediction for ~80% of the population or ~80% of the time for a given individual. Likewise, a word with 30% cloze probability would likely be the strongest prediction for ~30% of the population or ~30% of the time for a given individual. This means that the “most predictable word” would indeed be the participant’s current prediction (in that trial) in a larger proportion of the high constraint trials compared to the low constraint ones. Thus, a participant who encounters a large proportion of trials in which the most predictable word is presented in high constraint contexts will experience confirmation of their prediction more often than a participant who encounters a large proportion of trials in which the most predictable word is presented in low constraint contexts. It is therefore not possible to determine whether adaptation was triggered by the mere repeated confirmation of a participant’s prediction, or whether the strength of the confirmed prediction also played a role in the adaptation mechanism.

The current study thus aims to test whether adaptation is influenced by prediction strength. In order to do so, we focus on adaptation due to prediction failure (discouraging further prediction), rather than due to successful prediction (encouraging further prediction). This allows us to manipulate prediction strength independently of the predictability of the presented word, i.e., by presenting low cloze words in high vs. low constraint, we manipulate the strength of the initial prediction (strong or weak, respectively), while keeping the presented word equally unpredictable in both cases. In this way, we test whether adaptation is specifically triggered by unexpected words that appear in a context where an initially strong prediction could be generated (i.e., high constraint), relative to similarly unexpected words that appear in a context where no strong prediction was available (i.e., low constraint). Two experiments were conducted, in which the proportion of disconfirmed strong predictions was manipulated between participants, and we tested the influence of this proportion on prediction failure costs throughout the experiment. As stated above, our hypothesis was that disconfirmation of strong predictions serves as a trigger for adaptation, decreasing the tendency to commit to strong predictions in order to avoid prediction failure costs. If our hypothesis is correct, prediction failure costs should decrease as the experiment progresses, as the participants experience disconfirmation of strong predictions. Crucially, the greater the proportion of disconfirmed strong predictions a participant encounters, the more their prediction failure costs should be reduced, which should result in smaller prediction failure costs overall, as well as a greater rate of decrease in these costs throughout the experiment. In addition, we formulate a Bayesian adaptation model and show that it accounts for the trial-by-trial adaptation of prediction.

## Experiment 1

### Methods

The design and analyses for this study were pre-registered on the open science framework (OSF). The pre-registration report for Experiment 1 can be found at: https://osf.io/hwdq4/?view_only=516dcfb53b814d7483bdff03e61c271e. Data and analysis code can be found at: https://osf.io/d9s8g/?view_only=3123cc4830db42bc80ed31a5c5ed029f.

#### Participants

Participants were 120 Tel-Aviv University students (42 males), all native Hebrew speakers, with an average age of 24.33 (range: 18–36). Participants were given course credit or were paid 15 NIS (~4.5$) for their participation. The experiment was approved by the Ethics Committee at Tel Aviv University. Ten additional participants completed the experiment but were excluded from the analysis due to low accuracy in the task (the pre-registered exclusion criterion was below chance performance in either the congruent or the anomalous trials).

#### Materials

The materials were in Hebrew. They consisted of two-word phrases in which the first word was either highly constraining (i.e., had a highly probable completion) or not (i.e., did not have any highly probable completion), based on a cloze questionnaire (described below). The second word was always unexpected (i.e., a low cloze probability word), as determined by the cloze questionnaire results. This created two trial types: high constraint context – low cloze probability completion (High-Low, HL), and low constraint context – low cloze probability completion (Low-Low, LL). See Table 1 for example materials.

**Table 1:** Example materials for Experiment 1.

Trial type	First word	Second word	Second word with highest cloze probability (not presented in the experiment)
High constraint, Low cloze probability (HL)	*bu’ot* bubbles	*avir* air Cloze probability: 3.2% Translation of the phrase: “Air bubbles”	*sabon* soap Cloze probability: 93.5% Translation of the phrase: “Soap bubbles”
Low constraint, Low cloze probability (LL)	*šulxan*	*kafe* coffee Cloze probability: 3.0% Translation of the phrase: “Coffee table”	*oxel* food Cloze probability: 30.3% Translation of the phrase: “Dining table”

Twelve critical trials from each condition were presented to all participants. Filler trials were used in order to manipulate the proportion of HL and LL trials between participants: half of the participants encountered 72 additional HL trials, and half encountered 72 additional LL trials (see Table 2). The trials from each type (including the fillers) were distributed throughout the experiment in a pseudo-randomized order (different for each participant). Twenty-four anomalous filler items (e.g., “socks cake”) were also included, in order to enable the task (anomaly detection, see Procedure).

**Table 2:** Trial composition in each list in Experiments 1 and 2.

Experiment 1 (Hebrew)		Experiment 2 (English)		
Low-low list	High-low list	Low-low list	Mixed list	High-low list
		15 HH trials 3 Anomalies	15 HH trials 3 Anomalies	15 HH trials 3 Anomalies
12 HL critical trials 12 LL critical trials **72 LL filler trials** 24 Anomalies	12 HL critical trials 12 LL critical trials **72 HL filler trials** 24 Anomalies	12 HL critical trials 12 LL critical trials 12 HH critical trials **60 LL filler trials** 24 Anomalies	12 HL critical trials 12 LL critical trials 12 HH critical trials **30 HL filler trials** **30 LL filler trials** 24 Anomalies	12 HL critical trials 12 LL critical trials 12 HH critical trials **60 HL filler trials** 24 Anomalies

Presentation order of the trials listed in each cell of the table was pseudo-randomized for each participant (keeping each trial type evenly distributed). The trials that differ between lists (in each experiment) are marked in bold.

The LL and HL items were matched for length and frequency of the second word, overall (Length: HL mean = 4.66, LL mean = 4.89, *p* = 0.493, length was measured in number of letters; frequency: HL mean = 51.02, LL mean = 37.52, *p* = 0.519, frequency was taken from the corpus of Linzen, 2009), and for the 12 critical trials (Length: HL mean = 4, LL mean = 4.65, *p* = 0.191; frequency: HL mean = 30.67, LL mean = 17.83, *p* = 0.202). The critical trials were also matched for basic RTs for the second word, i.e., RTs in a lexical decision task for the second word in each item (without the presentation of the first word in the phrase) were similar in both conditions (HL mean = 578.84, LL mean = 579.07, *p* = 0.860). The basic RTs were collected from 20 participants, different from those in the main experiment.

Cloze probability questionnaires were conducted in order to assess constraint and cloze probability for each item. Participants (different from those in the main experiment) were presented with the first word of an item, and were instructed to provide the first completion that comes to mind. Each item was presented to 30–35 participants. Presentation order was randomized for each participant. High constraint items had a constraint of 65% or higher, low constraint items had constraint of 35% or lower. The average constraint was 83.03% in the high constraint items (87.03% in the 12 critical HL trials), and 24.51% in the low constraint items (19.82% in the 12 critical LL trials). HL and LL items were matched for cloze probability of the second word, with average cloze probability of 4.40% in the HL trials, and 4.46% in the LL trials, overall (*p* = 0.964), and in the 12 critical trials: 1.97 and 2.06% in the HL and LL trials, respectively (*p* = 0.865).

#### Procedure

Stimuli were presented using the E-prime 2.0 software (Psychology Software Tools, Pittsburgh, PA). Each trial was preceded by a 200 ms fixation cross. The two-word phrases were presented word-by-word in the middle of the screen. The first word was presented for 750 ms, with a 350 ms ISI. The second word was presented until the participant made a response, or up to 4 s (i.e., if the participant did not make a response within 4 s, the trial was terminated). Participants were instructed to press a green or a red button to indicate whether or not the phrase was congruent (respectively), as quickly as possible once the second word appears. Reaction times were recorded. After each trial, a string of hash keys (####) appeared on the screen and the participants pressed a button when they were ready to start the next trial. Prior to the experiment, participants completed a practice block of six trials.

#### Data Analysis

Reaction times were analyzed with linear mixed-effects models. Analyses were conducted using the lmerTest package (Kuznetsova et al., 2014) in the R software environment. Only the data from the critical trials was included in the initial analysis (data from all non-anomalous trials was included in the Bayesian adaptation model, see below). Trials with errors (i.e., trials in which the participant pressed the red button, indicating that the phrase is incongruent) were excluded. Outliers were trimmed by replacing data points exceeding 2.5 SDs from each participant’s mean with the value of 2.5 SDs from that participant’s mean (affecting 2.9% of the data). RTs were logarithmically transformed before being entered into the model. The model included the factors List (HL list and LL list, with LL list as the reference level), Trial type (HL and LL, with LL as the reference level), and Trial number (the position of the trial throughout the experiment). The binary factors (List and Trial type) were coded for simple contrasts (one level of the factor coded as 0.5, and the other as −0.5). All models initially included random intercepts for participants and items and were fully crossed (including all factors and their interaction as random slopes for items, and Trial type, Trial number, and their interactions as random slopes for participants; List was not included as random slope for participants since each participant belongs to only one level of this factor). However, all random slopes had to be removed in order to achieve convergence (this was done by iteratively removing the random slope associated with the smallest variance, Barr et al., 2013).

### Results

#### Accuracy

As mentioned above, the performance of all participants included in the analysis was above chance in both the congruent and the anomalous trials (separately). The average accuracy in the critical trials was 95.1% (SD = 4.30%), with high performance across conditions (LL list: LL trials – 99.2%, HL trials – 89.9%; HL list: LL trials – 98.6%, HL trials – 92.5%). Accuracy was analyzed using a logistic mixed-effects model, with the factors List (HL and LL, with LL list as the reference level) and Trial type (HL and LL, with LL as the reference level). There was an effect of Trial type such that accuracy was higher in the LL trials than in the HL trials (Estimate = −1.81, SE = 0.40, *z* = −4.54, *p* < 0.001). There was no significant effect of list (Estimate = 0.12, SE = 0.26, *z* = 0.47, *p* = 0.637), nor an interaction between Trial type and List (Estimate = 0.75, SE = 0.52, *z* = 1.45, *p* = 0.146).

#### Linear Regression Analysis: Pre-registered Analysis

**Figure 1:**
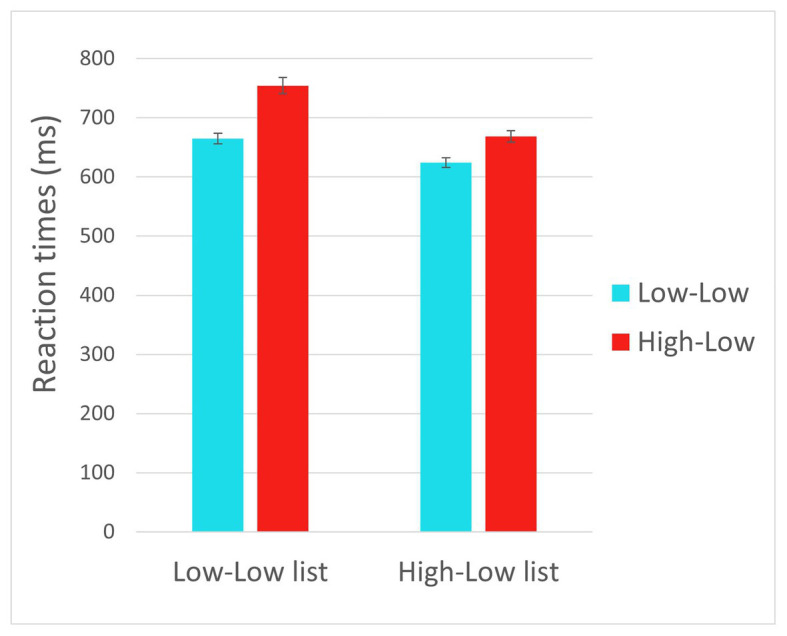
Reaction times in the critical trials in Experiment 1.

The full results of the analyses are reported in Table 3. Reaction times are displayed in Figure 1. The results (Model 1) showed an effect of Trial type such that RTs (for the critical trials) where longer for HL trials than for LL trials (*p* < 0.001), reflecting prediction failure costs. There was also an effect of List such that RTs were shorter in the HL list relative to the LL list (*p* = 0.002). These two effects were qualified by a significant interaction between List and Trial type, such that the difference between HL and LL trials was reduced in the HL list relative to the LL list (*p* = 0.048), indicating that frequent disconfirmation of strong predictions led to reduced prediction failure costs. There was also an effect of Trial number, such that RTs decreased as the experiment progressed (*p* < 0.001). Notably, we expected a three-way interaction between Trial type, List, and Trial number, indicating that throughout the experiment, the rate at which reaction times for HL trials decreased was greater for participants in the HL list than in the LL list. However, no interaction involving Trial number reached significance (see Discussion for a possible reason). We therefore formulated an adaptation model in order to capture the trial-by-trial dynamics.

**Table 3:** Mixed-effects regression models coefficients for Experiment 1.

	Estimate	SE	df	*t*-value	*p*-value
**Model 1**					
List	−0.0493	0.0160	219.6	−3.081	0.002^*^
Trial type	0.0440	0.0094	2,612	4.656	<0.001^*^
Trial number	−0.0006	0.0001	2,612	−9.069	<0.001^*^
List × Trial type	−0.0372	0.0189	2,611	−1.970	0.048^*^
List × Trial number	0.0002	0.0002	2,612	1.460	0.144
Trial type × Trial number	0.0001	0.0002	2,612	−0.980	0.327
List × Trial type × Trial number	0.0003	0.0003	2,612	1.057	0.290
**Model 2**					
Cloze probability	−0.0033	0.0006	166.4	−5.654	<0.001^*^
Inhibition index	0.0040	0.0002	10,610	18.738	<0.001^*^
**Model 3**					
List	−0.0179	0.0151	216.8	−1.190	0.235
Trial type	−0.0007	0.0107	907.1	−0.066	0.947
Trial number	−0.0004	0.00006	10,780	−5.797	<0.001^*^
Inhibition index	0.0027	0.0003	10,860	8.558	<0.001^*^
List × Trial type	0.0165	0.0147	10,880	1.110	0.267
List × Trial number	−0.00003	0.0001	10,740	−0.309	0.758
Trial type × Trial number	0.0003	0.0001	10,760	2.345	0.019^*^
List × Trial type × Trial number	−0.0002	0.0002	10,730	−0.989	0.323

* p < 0.05.

#### Bayesian Adaptation Model: Exploratory Analysis

In order to account for the trial-by-trial data, we formulated a Bayesian adaptation model whereby inhibition cost at each trial was modeled as μ*PE, such that:

1. μ is a point estimate for the participant’s belief about the likelihood of encountering the expected word (i.e., their current estimation of predictive validity). This value is defined as the mean of a beta distribution, updated on each trial, with an initial prior of beta(1, 1). Updating occurs whenever the participant encounters an HL trial: beta(1, 1 + number of HL trials encountered). This has the effect of lowering the estimated predictive validity with more encountered instances of failed prediction.
2. PE is the prediction error, defined as the difference between the constraint of the item and the cloze probability of the second word.

The inhibition index (μ*PE), reflecting inhibition costs for a trial, therefore is large: (i) when μ is large, i.e., the participant believes they will encounter the expected word (since they have not experienced many prediction failures); and/or (ii) when PE is large – the first word is highly constraining, and the second word is highly unpredictable.

**Figure 2:**
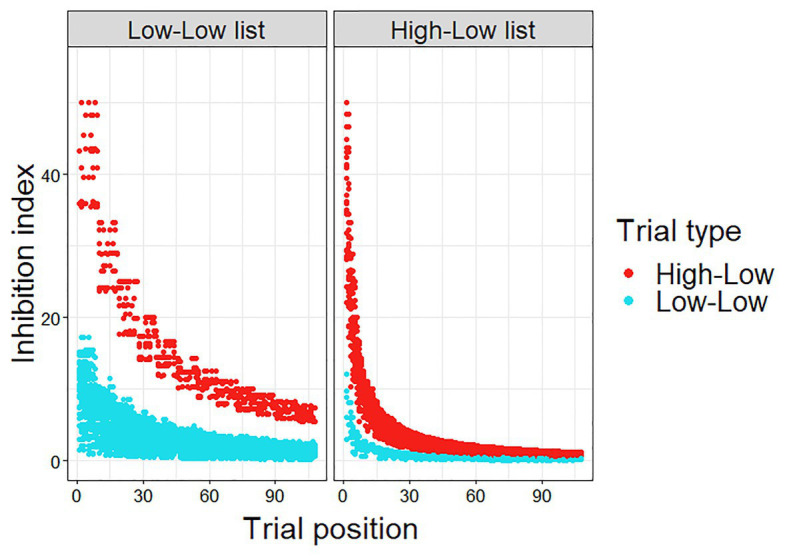
Calculated inhibition index (μ*PE) in Experiment 1.

The inhibition index was calculated for each trial, experimental and filler.^1^ As can be seen in Figure 2, the calculated inhibition index was higher for HL trials than for LL trials, since the prediction error is smaller in the LL trials. In addition, the calculated inhibition index decreases as the experiment progresses, as μ becomes smaller with the accumulation of more HL trials, and more so for the HL trials. Importantly, this decrease is greater and faster in the HL list, as in this list, which includes more HL trials, μ becomes smaller at a faster rate.

In order to test whether this inhibition index is a significant predictor of the data, we entered it into a linear mixed-effect regression. Note that the inhibition index only reflects the expected costs of prediction failure, but does not account for facilitatory effects of correct predictions. Namely, for a given HL or LL item, the majority of participants would not have predicted the low cloze word that was presented, and the costs associated with this scenario are modeled in the inhibition index. However, a portion of the participants (which correlates to the word’s cloze probability) would have predicted the presented word and would have therefore experienced facilitation, which is not accounted for by the inhibition index. To account for these facilitatory effects, we included the cloze probability of the presented second word as a predictor in the model, in addition to inhibition index (Model 2, see Table 3). The results showed that the inhibition index was a significant predictor of reaction times (*p* < 0.001).

Furthermore, the inhibition index was entered as an additional predictor in the initial model (Model 1 above) in order to test whether it is a significant predictor of reaction times above and beyond List, Trial type, and Trial number (Model 3; Table 3). The inhibition index remained a significant predictor of reaction times in this model (*p* < 0.001), indicating that it explains variance in reaction times beyond the original factors. The performance of the Bayesian adaptation model (Model 2) was also compared to alternative models, which include similar (or the same) information to the information that went into the calculation of the inhibition index, but as separate factors (i.e., without the assumptions of the adaptation model): (1) a model which included PE (the difference between constraint and cloze probability), Trial number, and the interaction between these factors. (2) A model which included PE, the number of HL trials encountered, and the interaction between these factors. The Bayesian adaptation model outperformed the alternatives (Bayesian model: AIC = −16,990, BIC = −16,903, Log likelihood = 8507.1; Alt1: AIC = −16,712, BIC = −16,712, Log likelihood = 8388.6; Alt2: AIC = −16,926, BIC = −16,874, Log likelihood = 8388.6; *p* < 0.001). These results indicate that the assumptions of the Bayesian adaptation model indeed increase its explanatory power, relative to models including the basic information entered into its calculations, but without its further assumptions. Namely, the calculation of the inhibition index increases the variance explained by the model, relative to models that include the same data but without this calculation.

### Discussion

The current experiment manipulated the proportion of disconfirmed strong predictions (HL trials) throughout the experiment, and tested the influence of this proportion on prediction failure costs. First, the results showed increased reaction times in the HL trials relative to LL trials. Since these conditions did not differ in the predictability of the second word in the phrase (i.e., cloze probability did not differ between these conditions), this result provides additional evidence for the incurrence of prediction failure costs (see General Discussion). Moreover, the results showed that this increase in reaction times in the HL relative to LL trials was smaller in the HL list than in the LL list, indicating that participants who experienced disconfirmation of strong predictions more often adapted to the experimental context by reducing their engagement in strong prediction. Since the filler items that differed between lists did not contrast in how predictable the presented words were (i.e., cloze probability), but only in the strength of the initially available prediction (i.e., constraint), this result supports our main hypothesis that the disconfirmation of strong predictions, rather than simply the occurrence of unpredictable words, triggers adaptation.

We additionally expected a three-way interaction between Trial type, List, and Trial number, reflecting that throughout the experiment the rate at which reaction times for HL trials decreased was greater for participants in the HL list than in the LL list. However, we did not find this interaction. We believe, based on examination of the data that adaptation in the HL list occurred too quickly to be detectable in our experiment. The proportion of HL trials in the HL list was very high – seven HL trials for every LL and anomaly trial. In addition, the experiment did not include high constraint trials in which the predicted word appeared. Given this, adaptation, namely learning that strong predictions are extremely likely to be disconfirmed in the experiment, may have taken place prior to any critical trials, or after very few of them.

In the absence of the predicted three-way interaction, in order to better account for the trial-by-trial dynamics, we formulated a Bayesian adaptation model. We showed that this model, which takes into consideration the ongoing updating of the participant’s belief about the likelihood of encountering a predictable word (i.e., their estimate of predictive validity), can capture the data.

## Experiment 2

In Experiment 1, the Bayesian model and the related analyses were conceived after data collection, and were thus exploratory. Since the addition of unplanned analyses greatly increases the likelihood of false positives, we then followed up with a replication experiment (Experiment 2), for which the Bayesian model and related analyses were pre-registered. In this experiment, we also included high constraint – high cloze probability (HH) trials, in an attempt to slow down adaptation. The Bayesian adaptation model was therefore extended to include such trials (see below). Additionally, in this experiment we included three lists (instead of two), in order to manipulate the proportion of HL trials more gradually.

In addition, while Experiment 1 was a lab-based experiment with Hebrew speakers, Experiment 2 was in English, conducted online with native English speakers. This was done due to considerations of participant recruitment, and was not predicted to affect the results. However, the use of new materials in a different language, and a different participant population, does contribute to the generalizability of our findings.

### Methods

The design and analyses for this study were pre-registered on the OSF. The pre-registration report for Experiment 2 can be found at: https://osf.io/3k6am/?view_only=2bd9dc5c43c2459385bead7cf03978f6. Data and analysis code can be found at: https://osf.io/5h9tv/?view_only=c2f47d6d3adf405297b1c863b88b3818.

#### Participants

Participants were 150 (69 males) native English speakers, born and living in the United States, with an average age of 31.11 (range: 20–45). The participants were recruited *via* Prolific and were paid 1.5 GBP (~2$) for their participation. The experiment was approved by the Ethics Committee in Tel Aviv University. Fourteen additional participants completed the experiment but were excluded from the analysis: 12 due to low accuracy in the task, and two due to mean RTs that exceeded 2.5 SD from the group’s mean RT (based on the pre-registered exclusion criteria).

#### Materials

As in Experiment 1, the materials included 12 HL and 12 LL critical trials that were presented to all participants. Additionally, 12 high constraint, high cloze probability (HH) critical trials were included. Constraint and cloze probability were determined based on a cloze questionnaire, as described below. See Table 4 for example materials. The HH items were introduced in the current experiment in order to slow down adaptation, by indicating to the participant that predictions can be confirmed in the experimental context. Filler trials were manipulated between participants, such that one third of the participants encountered 60 additional HL trials, one third encountered 60 additional LL trials, and one third encountered 30 additional HL trials and 30 additional LL trials. The different trial types were distributed throughout the experiment in a pseudorandomized order. However, 15 additional HH trials were presented to all participants at the beginning of the experiment, in order to make sure all participants could initially assume that forming predictions is beneficial in the experimental context. Twenty-four anomalous filler items (e.g., “socks cake”) were also included, in order to enable the task (anomaly detection, see Procedure). The trial composition in each list is summarized in Table 2.

**Table 4:** Example materials for Experiment 2.

Trial type	First word	Second word	Second word with highest cloze probability (not presented in the experiment in HL and LL trials)
High constraint, Low cloze probability (HL)	Rearview	camera Cloze probability: 6.7%	mirror Cloze probability: 93%
Low constraint, Low cloze probability (LL)	Desert	storm Cloze probability: 6.8%	island Cloze probability: 14%
High constraint, High cloze probability (HH)	Peanut	butter Cloze probability: 83%	

The LL and HL items were matched for length and frequency of the second word, overall (Length: HL mean = 6.05, LL mean = 6.23, *p* = 0.591, length was measured in number of letters; frequency: HL mean = 78.03, LL mean = 92.70, *p* = 0.470, frequency was taken from the Corpus of Contemporary American English, COCA, Davies, 2009). The LL, HL, and HH items were matched for length and frequency of the 12 critical trials (Length: HL mean = 5.59, LL mean = 6.58, HH mean = 5.75, HL vs. LL: *p* = 0.312, HH vs. LL: *p* = 0.791, HH vs. HL: *p* = 0.842; frequency: HL mean = 100.76, LL mean = 86.80, HH mean = 113.7, HL vs. LL: *p* = 0.450, HH vs. LL: *p* = 0.780, HH vs. HL: *p* = 0.789).

Cloze probability questionnaires were conducted in order to assess constraint and cloze probability of each item. Each item was presented to 30 participants (different from those in the main experiment). Presentation order was randomized for each participant. High constraint items had a constraint of 50% or higher and low constraint items had a constraint of 25% or lower. The average constraint was 73.13% in the high constraint items (76.94% in the 12 critical HL trials, 72.48% in the 12 critical HH trials), and 14.64% in the low constraint items (14.44% in the 12 critical LL trials). HH and HL items were matched for constraint (*p* = 0.321). HL and LL items were matched for cloze probability (*p* = 0.450 overall, *p* = 0.316 for the critical items), with average cloze probability of 3.28% in the HL trials, and 2.73% in the LL trials (in the 12 critical trials: 6.94 and 4.72% in the HL and LL trials, respectively).

#### Procedure and Data Analysis

The procedure was as detailed for Experiment 1, except that the experiment was built in PsychoPy 2 (Peirce et al., 2019) and was run online on the Pavlovia platform.^2^ Data analysis was identical to Experiment 1, except that the factor Trial type included HH trials (i.e., HH, HL, and LL, coded for simple contrasts, with LL as the baseline level), and the factor List included three levels rather than two (this factor was treated as ordinal/continuous, since the three levels of this factor are ordered on a scale of the proportion of HL trials; thus, the three levels were included as one numerical variable: LL list = 1, mixed list = 2, and HL list = 3).

### Results

#### Accuracy

As mentioned above, the performance of all participants included in the analysis was above chance in both the congruent and the anomalous trials (separately). The average accuracy in the critical trials was 96.7% (SD = 2.72%), with performance high across conditions (LL list: HH trials – 99.7%, LL trials – 98.2%, HL trials – 90.0%; Mixed list: HH trials – 99.3%, LL trials – 99.2%, HL trials – 93.2%; HL list: HH trials – 99.2%, LL trials – 98.8%, HL trials – 93.0%). Accuracy was analyzed using a logistic mixed-effects model, with the factor Trial type (HH, LL and HL, with LL as the reference level) and List (HL, Mixed, LL, as an ordinal variable). There were effects of Trial type such that accuracy was higher in the HH trials than in the LL trials (Estimate = 2.87, SE = 1.08, *z* = 2.66, *p* = 0.008), and higher in the LL trials than in the HL trials (Estimate = −1.16, SE = 0.44, *z* = −2.63, *p* = 0.009). Additionally, there was an interaction between List and Trial type at the levels of HH vs. LL, such that the difference in accuracy between the HH and LL trials was smaller the higher the proportion of HL trials was (Estimate = −0.97, SE = 0.43, *z* = 2.27, *p* = 0.023). There was no significant effect of List (Estimate = 0.15, SE = 0.15, *z* = 0.97, *p* = 0.332), and the difference in accuracy between HL and LL trials did not differ significantly between lists, (Estimate = −0.24, SE = 0.21, *z* = −1.14, *p* = 0.255).

#### Linear Regression Analysis (Pre-registered)

**Figure 3:**
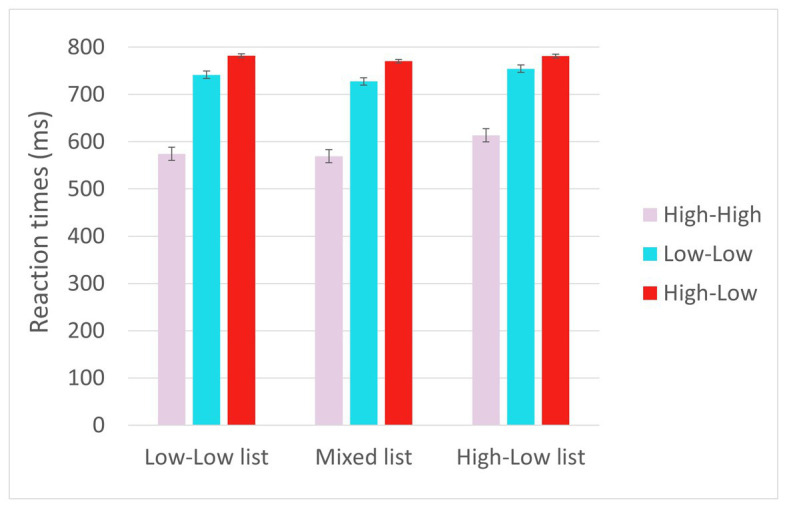
Reaction times in the critical trials in Experiment 2.

The full results of the analyses are reported in Table 5. Reaction times are displayed in Figure 3. The results (Model 1) showed effects of Trial type such that RTs (for the critical trials) were shorter for HH trials than for LL trials (*p* < 0.001), reflecting facilitation due to higher predictability in the HH trials; and longer for HL trials than for the LL trials (*p* < 0.001), and reflecting prediction failure costs. Additionally, there was a significant interaction between List and Trial type at the levels of HL vs. LL, such that the difference between HL and LL trials decreased the more HL trials the list included (*p* = 0.012). There was also an effect of Trial number, such that RTs decreased as the experiment progressed (*p* < 0.001), as well as an interaction between Trial number and Trial type at the levels of HL vs. LL such that the decrease in RTs as the experiment progressed was greater for HL trials than for LL trials (*p* = 0.011). Again, the three-way interaction between Trial type (HL vs. LL), List and Trial number did not reach significance.

**Table 5:** Mixed-effects regression models coefficients for Experiment 2.

	Estimate	SE	df	*t*-value	*p*-value
**Model 1**					
List	0.0017	0.0074	204.1	0.230	0.818
Trial type (HH vs. LL)	−0.1228	0.0195	1,075	−6.308	<0.001^*^
Trial type (HL vs. LL)	0.0635	0.0158	4,193	4.035	<0.001^*^
Trial number	−0.0005	0.0001	13,520	−4.928	<0.001^*^
List × Trial type (HH vs. LL)	−0.0004	0.0079	13,590	−0.054	0.957
List × Trial type (HL vs. LL)	−0.0169	0.0067	13,740	−2.516	0.012^*^
List × Trial number	0.0001	0.00004	13,520	1.568	0.117
Trial type (HH vs. LL) × Trial number	0.00003	0.0002	13,520	0.112	0.911
Trial type (HL vs. LL) × Trial number	0.0005	0.0002	13,520	−2.538	0.011
List × Trial type (HH vs. LL) × Trial number	0.0001	0.0001	13,520	1.073	0.283
List × Trial type (HL vs. LL) × Trial number	0.0002	0.0001	13,520	1.740	0.082
**Model 2**					
Cloze probability	−0.1349	0.0149	137.9	−9.034	<0.001^*^
Inhibition index	0.1303	0.0105	1,471	12.446	<0.001^*^
**Model 3**					
List	0.0061	0.0075	208.5	0.824	0.411
Trial type (HH vs. LL)	−0. 1,058	0.0183	873.8	−5.796	<0.001^*^
Trial type (HL vs. LL)	−0.0286	0.0218	1,564	−1.307	0.191
Trial number	−0.0004	0.0001	12,780	−4.134	<0.001^*^
Inhibition index	0.1573	0.0265	255.2	5.931	<0.001^*^
List × Trial type (HH vs. LL)	−0.0014	0.0079	13,610	−0.177	0.859
List × Trial type (HL vs. LL)	−0.0058	0.0071	2,578	−0.827	0.408
List × Trial number	0.0001	0.00004	12,930	2.342	0.019^*^
Trial type (HH vs. LL) × Trial number	0.00001	0.0002	13,490	0.039	0.969
Trial type (HL vs. LL) × Trial number	−0.0003	0.0002	10,330	−1.643	0.101
List × Trial type (HH vs. LL) × Trial number	0.0001	0.0001	13,520	0.883	0.377
List × Trial type (HL vs. LL) × Trial number	0.0002	0.0001	11,150	2.444	0.015^*^

* p < 0.05.

#### Bayesian Adaptation Model (Pre-registered)

**Figure 4:**
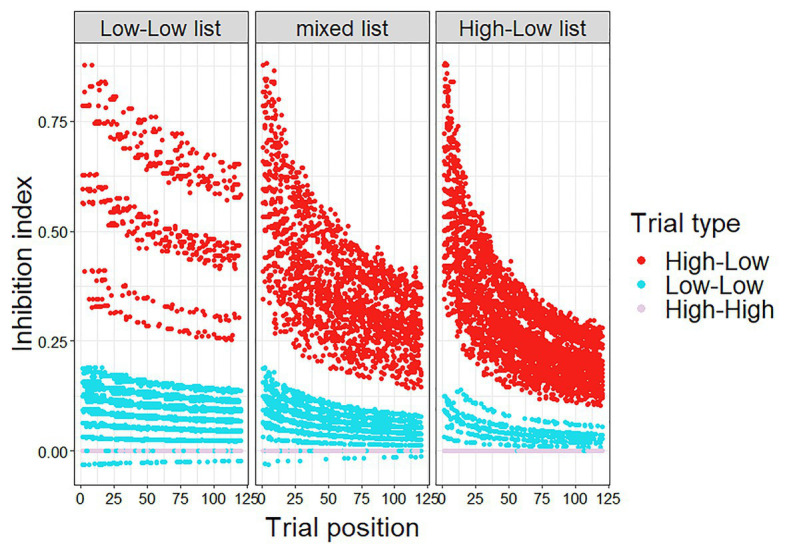
Calculated inhibition index (μ*PE) in Experiment 2.

The Bayesian adaptation model was similar to that of Experiment 1, modified for the inclusion of HH trials. Thus, in the current model, updating of the participant’s belief about predictive validity occurred whenever the participant encountered a high constraint trial, such that a HL trial lowered the estimated predictive validity (as in Experiment 1), and a HH trail raised the estimated predictive validity: beta(1 + number of HH trials encountered, 1 + number of HL trials encountered). The inhibition index (μ*PE) was calculated for each trial (Figure 4), and entered into a linear mixed-effect regression with cloze probability as an additional predictor (Model 2). The results showed that the inhibition index was a significant predictor of reaction times (*p* < 0.001).

The inhibition index was then entered as an additional predictor in the initial model (Model 1 above) in order to test whether it is a significant predictor of reaction times above and beyond List, Trial type, and Trial number (Model 3). The inhibition index remains a significant predictor of reaction times in this model (*p* < 0.001), indicating that it explains variance in reaction times beyond the original factors. Again, the performance of the Bayesian adaptation model (Model 2) was also compared to alternative models, which include similar (or the same) information to the information that went into the calculation of the inhibition index, but as separate factors (i.e., without the assumptions of the adaptation model): (1) A model which included PE (the difference between constraint and cloze probability), Trial number, and the interaction between these factors. (2) A model which included PE, the number of HL trials encountered, the number of HH trials encountered, and the interaction between these factors. The Bayesian adaptation model outperformed the alternatives (Bayesian model: AIC = −20,685, BIC = −20,549, Log likelihood = 10,360; Alt1: AIC = −20,623, BIC = −20,540, Log likelihood = 10,323; Alt2: AIC = −20,589, BIC = −20,537, Log likelihood = 10,302; *p* < 0.001), indicating that the assumptions of the Bayesian adaptation model indeed increase its explanatory power, relative to other models including the basic information entered into its calculations, but without its additional assumptions.

### Discussion

Experiment 2 replicated and extended the results of Experiment 1. First, the results showed increased reaction times in the HL trials relative to LL trials, providing additional evidence for the incurrence of prediction failure costs. In addition, the results showed that this increase in reaction times in the HL relative to LL trials was smaller the more HL trials the participant encountered, providing additional evidence that participants who encounter the disconfirmation of strong predictions more often adapt by reducing their engagement in strong prediction. This result thus provides additional support for our main hypothesis that the disconfirmation of strong predictions, rather than simply the occurrence of unpredictable words, triggers adaptation.

The Bayesian adaptation model was again shown to capture the trial-by-trial data, corroborating the results of the exploratory analysis in Experiment 1. Importantly, in Experiment 2 this model and the related analyses were pre-registered, alleviating the increased risk of false positives in an exploratory analysis.

## General Discussion

In the current study, we hypothesized that the disconfirmation of strong predictions serves as a trigger for adaptation, influencing subsequent processing by decreasing the participant’s tendency to commit to strong predictions, in order to avoid prediction failure costs. This hypothesis was tested in two experiments by manipulating the proportion of disconfirmed strong predictions encountered during the experiment and measuring the influence of this proportion on prediction failure costs.

First, the results of both experiments showed increased reaction times in trials consisting of a highly constraining word followed by an unpredictable word (HL trials), relative to trials where an unpredictable word appeared after a word which was not constraining (LL trials). Since these conditions did not differ in the predictability of the second word in the phrase (i.e., cloze probability did not differ between these conditions), this result provides evidence for prediction failure costs, i.e., costs that are incurred due to the initially formed prediction rather than due to the processing of an unpredictable word, in and of itself. This result is particularly interesting in light of recent evidence regarding the f-PNP ERP component. As discussed in the Introduction, prediction failure costs were often demonstrated in ERP studies showing a late frontal positivity (f-PNP) elicited by unexpected words only in high constraint contexts (e.g., Federmeier et al., 2007). However, in a recent study, Brothers et al. (2020) have tested the effect of context length on the f-PNP component. Their results showed a significant f-PNP effect elicited by unpredictable words in high constraint contexts, only when the context was rich and globally constraining, but not when the strong lexical prediction could only be generated based on a single word immediately preceding the target word. For example, the f-PNP was not observed in a sentence such as “(…) James unlocked the… door/laptop,” when constraint was purely reliant on a single word (“unlocked”). Similarly, a f-PNP was not observed by Lau et al. (2016), with materials consisting of a one-word context (a prenominal adjective). These results may thus suggest that impoverished contexts do not give rise to prediction failure costs, which is seemingly inconsistent with our current results, demonstrating prediction failure costs in two-word phrases (i.e., single word contexts). Crucially, however, there are several factors in the current materials and design, which may reconcile the current results with the results of Brothers et al. (2020). First, in the current study, we used a relatively slow presentation rate (the SOA was 1,000 ms, while the SOA in the experiments of Brothers et al., 2020, was 550 ms). The long SOA provided participants with more time to form strong and specific predictions (see e.g., Ito et al., 2016), which may have contributed to the incurrence of prediction failure costs. Additionally, the task in the current study was a speeded anomaly judgment task, while participants in the Brothers et al. (2020) study discussed above read for comprehension and then gave a non-speeded judgment, and participants in the Lau et al. (2016) study were not required to provide any response during the trials (a memory recognition test was administered after each block). Thus, the task in the current study may have provided further encouragement to generate predictions, in order to respond as quickly as possible once the second word appeared. Indeed, the f-PNP component was shown to be greater when prediction is encouraged by task demands (Brothers et al., 2017). Moreover, the average constraint in the current study was relatively high (87% in Exp. 1 and 77% in Exp.2, compared to 63% in the minimal context materials of Brothers et al., 2020), which could have significant influence on prediction failure costs, considering that the f-PNP component is only elicited in high constraint contexts. Thus, while the use of two-word phrases in the current study perhaps had some diminishing influence on prediction failure costs, the other factors discussed above may have outweighed this influence, allowing the manifestation of prediction failure costs nonetheless.

Importantly, the results also showed that this increase in reaction times in the HL relative to LL trials was smaller the higher the proportion of HL trials was in the experiment, indicating that participants who experienced disconfirmation of strong predictions more often adapted by reducing their engagement in strong prediction. Since the lists did not differ in how predictable the presented words were (i.e., cloze probability), but only in the strength of the initially available predictions (i.e., constraint), this result supports our main hypothesis that the disconfirmation of strong predictions, rather than simply the occurrence of unpredictable words, triggers adaptation.

We formulated a Bayesian adaptation model in order to account for the trial-by-trial adaptation dynamics. In this model, the comprehender iteratively updates their belief about predictive validity in the current situation. The comprehender’s estimate of predictive validity decreases when an unexpected word appears in a high constraint context (i.e., a HL trial), and increases when the predictable word appears in a high constraint context (i.e., a HH trial). This estimate of predictive validity is then used to weigh the strength of the subsequent prediction, thus alleviating prediction failure costs when the comprehender believes predictive validity is low and it is not beneficial to engage in strong prediction. This model was shown to be a significant predictor of reaction times in both experiments, first in an exploratory analysis in Experiment 1, and then in a pre-registered analysis in Experiment 2.

As discussed in the Introduction, processing resources are known to be limited and prediction can be considered a “wasteful” processing strategy, requiring the generation of predictions and the handling of disconfirmed predictions. The current study provides support for the notion that processing resources are nonetheless allocated efficiently, in that prediction is not always employed to the same extent. Instead, when situations differ in how beneficial prediction is, comprehenders rationally adapt their processing strategies, to increase or decrease the reliance on strong predictions.

### Prior Beliefs About Predictive Validity

In the current study, the main aim of the Bayesian model was to account for adaptation by modeling the change in participants’ beliefs about predictive validity throughout the experiment, and its influence on processing prediction failure. Although our focus was on changes in the estimated predictive validity, the model had to include an initial prior, representing the participant’s expected predictive validity when they arrive at the experiment, prior to any trials. The prior that we chose, beta(1,1), implies that the participant begins the experiment with a belief that the predictive validity is 50%, i.e., when encountering a predictive first word (a high constraint item) there is a 50% chance that the predicted word will be presented. This is not necessarily an accurate assumption. However, we chose to use this standard prior since determining a more accurate prior requires non-trivial decisions on parameters that we cannot assess. Essentially, the participants’ estimate of predictive validity at the beginning of the experiment should reflect the predictive validity in their accumulated linguistic experience, i.e., the likelihood of encountering the predicted word following a high constraint context. Namely, the prior should match the mean constraint of “high constraint” contexts in the language. However, we do not know the distribution of constraint in the language. Moreover, we do not know what constitutes a “high constraint” context. That is, while, we do believe that there is a qualitative difference in the processing of high and low constraint contexts (see section “The role of prediction failure in adaptation” below), we do not know where the threshold between the two lies. Thus, we cannot achieve a better estimation for the participants’ belief about predictive validity at the beginning of the experiment.

Additionally, we chose a weak prior (reflected in the sum of the two parameters to the beta distribution), since we assume that when participants approach an experimental task, they are relatively “prone to adaptation.” When engaging in conversation in everyday life it is reasonable for a comprehender to be relatively confident that they can rely on their previous experience, and they are therefore likely to give more weight to previous experience and need more evidence in order to adapt. In contrast, an experimental setting is either a new situation for the participant (for inexperienced participants) or a situation which the participant knows varies significantly between occurrences (i.e., upcoming input in a new experiment is not expected to resemble previous, unrelated, experiments that the participant may have participated in). Therefore, participants are likely not to put a lot of weight on their prior belief (i.e., have a weak initial prior).

It may be interesting to consider the influence that alternative priors would have on the output of the model. A prior which represents a higher initial estimate of predictive validity would result in a greater decrease in the estimated predictive validity with every HL trial encountered early in the experiment, leading to even faster adaptation than the current model predicts. Of course, lower initial estimates of predictive validity would have the opposite effect (i.e., slower adaptation). Additionally, the higher the weight of the initial prior, the slower the adaptation would be, since more evidence would be needed in order to outweigh previous experience. Although it is possible to try and determine the initial prior that would provide the best fit for the current data, we did not explore this issue further in this study, as this prior would mostly indicate how participants approach the experimental situation, and is not necessarily generalizable to real-life situations. Importantly, these considerations about the initial prior are orthogonal to our main aim and conclusions in the current paper, since we manipulated the proportion of disconfirmed strong predictions between lists, and participants were randomly assigned a list, i.e., there is no ground to assume a systematic difference between lists in the initial prior participants arrive with.

### The Role of Prediction Failure in Adaptation

The current results provide evidence for the importance of prediction failure as a trigger for adaptation of prediction. Namely, the manipulation in the current study was achieved by presenting either HL fillers, or LL fillers (or both), which differ in constraint but not in cloze probability. Thus, the adaptation, we observed is driven by prediction failure, i.e., by the disconfirmation of highly probable predictions. This conclusion accords with the prevalent notion that prediction errors are crucial for implicit learning, as they signal the need to update future predictions (e.g., Shanks, 1995; Schultz et al., 1997; Schultz and Dickinson, 2000). A basic principle in numerous learning/adaptation models, inherent to prominent frameworks such as reinforcement learning and Bayesian adaptation, is that the extent of learning/adaptation exerted by a given input depends on the prediction error experienced. For example, Jaeger and Snider (2013) have shown that syntactic alignment increases as a function of the prediction error experienced, while processing the prime structure, i.e., the same syntactic structure can exert stronger or weaker syntactic priming depending on how surprising it was when it appeared as a prime. Notably, their results show that the extent of adaptation depends on both prior and recent experience. Specifically, they show that syntactic alignment is stronger when the prime’s structure is unexpected given the verb’s bias (i.e., when prediction error is large based on prior experience), but also when the prime’s structure was infrequent in previous trials in the experiment (i.e., when prediction error is large based on recent experience). The influence of both prior and recent experience on the extent of adaptation is also evidenced in the current study, and implemented in our adaptation model. First, HL trials, in which the participant can experience a significant prediction error, induce adaptation, while LL trials do not. This is an influence of prior experience, i.e., a low cloze word in a high constraint context incurs larger prediction error than in a low constraint context, based on the participant’s accumulated knowledge regarding the cloze probability distribution (or some representation of it). Additionally, in a Bayesian adaptation model, the more improbable an input is given the prior, the greater the update it causes. This is implemented in the calculation of the participant’s estimated predictive validity (*μ*) in our model: a HL trial encountered early in the experiment, when the estimated predictive validity is higher, induces a greater change to the participant’s belief about predictive validity (and thus a greater change to the behavior in subsequent trials) than a HL trial encountered later in the experiment, when the estimated predictive validity is lower (and vice versa for a HH trial). This is an influence of recent experience, i.e., despite the participant’s prior knowledge regarding the cloze probability distributions, the prediction error experienced when a low cloze word appears in a high constraint context has less of an effect as the participant learns not to expect the high cloze word.

We note that although in the current study, we take the approach of formulating a Bayesian adaptation model, and the results show that this model accounts for reaction times in our experiments, the same data can potentially be compatible with models based on other frameworks (e.g., reinforcement learning). However, the choice to model Bayesian adaptation is motivated by the vast literature employing such models to account for a myriad of phenomena in different domains, such as formal semantics (e.g., Lassiter and Goodman, 2015), reasoning (e.g., Heit, 1998), Bayesian pragmatics (e.g., Werning et al., 2019), and, most relevantly, priming effects in language processing (e.g., Myslín and Levy, 2016; Delaney-Busch et al., 2019). Importantly, the performance of the Bayesian adaptation model in the current study indicates that any model that would account for the data should implement the basic notions that adaptation is initiated by the incompatibility of the input with the participant’s predictions (i.e., prediction error) and that the extent of adaptation at each trial is dependent on how incompatible the trial is with the predictions generated, which leads to the non-linear adaptation throughout the experiment (i.e., greater adaptation in earlier trials).

### Pre-updating, Commitment, and Inhibition

The current results provide additional evidence indicating that prediction failure costs can be influenced by adaptation (as also demonstrated by Schwanenflugel and Shoben, 1985, see Introduction). This raises the question of how prediction failure costs are reduced, i.e., which process (or processes) is made easier, or is even eliminated, when adaptation occurs.

As discussed in the Introduction, prediction failure costs were suggested to stem from a need to inhibit the falsely predicted word due to commitment made to the strong prediction (e.g., Ness and Meltzer-Asscher, 2018a,b). This commitment was recently suggested to be the result of a prediction mechanism termed “pre-updating” (Lau et al., 2013; Kuperberg and Jaeger, 2016; Ness and Meltzer-Asscher, 2018b), which involves not only the activation of the predicted content, but its actual integration into the sentence’s representation being built in working memory. Since a pre-updated prediction is integrated into the sentence representation, if it is then disconfirmed, inhibition is required in order to “override” the integrated representation and allow integration of the actual input instead. Interestingly, overriding an integrated representation may require inhibition or suppression at different levels of representation (Kuperberg et al., 2020). Ultimately, the high-level representation of the sentence or the event being conveyed by the sentence (and preceding context) needs to be corrected to no longer include the wrong prediction. This correction of the high-level representation entails suppression of the incorrectly predicted event, and may or may not require inhibition of the lower-level representation of the predicted word or its semantic features. Indeed, recent experiments employing the cross-modal lexical priming (CMLP) paradigm provided indication that inhibition of the wrongly predicted word can be observed when a (congruent) unexpected word is presented in a highly constraining sentence, and that this inhibition may be correlated with the f-PNP component (Ness and Meltzer-Asscher, 2018a). Thus, prediction failure costs (and the f-PNP component) may encompass processes at multiple levels of representation.

Due to these costly processes that are needed when a pre-updated prediction is disconfirmed, pre-updating constitutes a strong form of prediction, which can occur only when a highly probable (highly pre-activated) prediction is available. Pre-updating was recently suggested to be initiated by an activation threshold, i.e., when the activation level of a predicted word passes a threshold, this word will be pre-updated (Ness and Meltzer-Asscher, 2018b, 2021). Thus, we propose that the underlying mechanism by which prediction failure costs are modulated is the adjustment of the threshold for pre-updating. When the estimated predictive validity is decreased, the threshold for pre-updating is raised, leading to a lower tendency to pre-update. In such a situation, when pre-updating is avoided, the disconfirmation of a high cloze prediction would not require inhibition, alleviating prediction failure costs. In the opposite situation, when the estimated predictive validity is increased, the threshold is lowered, leading to a higher tendency to pre-update. In such a situation, if a strong prediction is then disconfirmed, prediction failure costs will be increased, since the disconfirmed prediction is more likely to have been pre-updated, requiring inhibition when revealed not to be correct.

## Conclusion

As discussed in the introduction, the current study aimed at addressing two questions regarding the adaptation of prediction. First, what triggers it; and second, which aspects of prediction are adaptable. The current study addressed these questions with regard to prediction failure, providing evidence that prediction failure can serve as a trigger for adaptation, and that prediction failure costs are adaptable (i.e., can be influenced by adaptation). We show that a Bayesian adaptation model can account for the trial-by-trial dynamics, and propose that the adaptation of prediction failure costs is achieved *via* a thresholding mechanism adjusting the tendency to commit to strong predictions.

## Data Availability Statement

The datasets presented in this study can be found in online repositories. The names of the repository/repositories and accession number(s) can be found at: OSF: https://osf.io/d9s8g/?view_only=3123cc4830db42bc80ed31a5c5ed029f and https://osf.io/5h9tv/?view_only=c2f47d6d3adf405297b1c863b88b3818.

## Ethics Statement

The studies involving human participants were reviewed and approved by the Ethics Committee at Tel Aviv University. The patients/participants provided their written informed consent to participate in this study.

## Author Contributions

TN and AM-A contributed to the conceptualization and design of the study, and the writing of the manuscript. TN conducted the experiments and the analyses. AM-A supervised and provided funding and resources. All authors contributed to the article and approved the submitted version.

### Conflict of Interest

The authors declare that the research was conducted in the absence of any commercial or financial relationships that could be construed as a potential conflict of interest.

## Footnotes

^1^We note that the inhibition index reflects the expected cost of inhibition, which we expect to only take place in (and be affected by) high constraint trials. However, for practical reasons, we had to decide how to handle LL trials in the analyses, which include the inhibition index. Treating the value of the inhibition index for all LL trials as missing value was not possible, since this is not a situation of “missing at random” (i.e., there would be a systematic difference between trials with a “missing” inhibition index value and trials with actual values), which would distort the regression results. For consistency, we therefore chose to have a uniform formula for the calculation of all trials, with the assumption that the inhibition index for LL trials would not contribute much to the explanatory power of the model in any case, as it is low and relatively invariable (due to small prediction error). The alternative would be to set the value of the inhibition index to zero in all LL trials, representing the lack of prediction failure and no inhibition. In order to ensure that our results and conclusions do not hinge on the decision to compute an inhibition index for LL trials rather than set it to zero, we ran the analyses for both experiments again, but with the inhibition index set to zero in all LL trials. This modification had very little effect on the results. Crucially, none of the significant results in the original analyses became non-significant or vice versa.

^2^pavlovia.org
